# Evaluation of the neighborhood environment walkability scale in Nigeria

**DOI:** 10.1186/1476-072X-12-16

**Published:** 2013-03-21

**Authors:** Adewale L Oyeyemi, James F Sallis, Benedicte Deforche, Adetoyeje Y Oyeyemi, Ilse De Bourdeaudhuij, Delfien Van Dyck

**Affiliations:** 1 Department of Physiotherapy, College of Medical Sciences, University of Maiduguri, Maiduguri, Nigeria; 2 Department of Movement and Sports Sciences, Faculty of Medicine and Health Sciences, Ghent University, Ghent, Belgium; 3 Department of Family & Preventive Medicine, University of California, San Diego, USA; 4 Department of Biometry and Biomechanics, Faculty of Physical Education and Physiotherapy, Vrije Universiteit Brussels, Brussels, Belgium; 5 Research Foundation Flanders, Brussels, Belgium

**Keywords:** Built environment, Physical activity, Measurements, Psychometric, Africa

## Abstract

**Background:**

The development of reliable and culturally sensitive measures of attributes of the built and social environment is necessary for accurate analysis of environmental correlates of physical activity in low-income countries, that can inform international evidence-based policies and interventions in the worldwide prevention of physical inactivity epidemics. This study systematically adapted the Neighborhood Environment Walkability Scale (NEWS) for Nigeria and evaluated aspects of reliability and validity of the adapted version among Nigerian adults.

**Methods:**

The adaptation of the NEWS was conducted by African and international experts, and final items were selected for NEWS-Nigeria after a cross-validation of the confirmatory factor analysis structure of the original NEWS. Participants (N = 386; female = 47.2%) from two cities in Nigeria completed the adapted NEWS surveys regarding perceived residential density, land use mix – diversity, land use mix – access, street connectivity, infrastructure and safety for walking and cycling, aesthetics, traffic safety, and safety from crime. Self-reported activity for leisure, walking for different purposes, and overall physical activity were assessed with the validated International Physical Activity Questionnaire (long version).

**Results:**

The adapted NEWS subscales had moderate to high test-retest reliability (ICC range 0.59 –0.91). Construct validity was good, with residents of high-walkable neighborhoods reporting significantly higher residential density, more land use mix diversity, higher street connectivity, more traffic safety and more safety from crime, but lower infrastructure and safety for walking/cycling and aesthetics than residents of low-walkable neighborhoods. Concurrent validity correlations were low to moderate (r = 0.10 –0.31) with residential density, land use mix diversity, and traffic safety significantly associated with most physical activity outcomes.

**Conclusions:**

The NEWS-Nigeria demonstrated acceptable measurement properties among Nigerian adults and may be useful for evaluation of the built environment in Nigeria. Further adaptation and evaluation in other African countries is needed to create a version that could be used throughout the African region.

## Introduction

Physical inactivity is the fourth leading cause of death worldwide, with most of these deaths occurring in developing countries [1-3]. Increasing physical activity is one of four strategies recommended by the United Nations to reduce global epidemics of non-communicable diseases (NCD) [4]. The World Health Organization [5] and numerous authoritative groups [6-10] recommended environmental and policy interventions as viable strategies to improve physical activity and prevent obesity worldwide. The theoretical framework for understanding such interventions is the ecological model that is based on the principle that health behaviors are influenced by multiple levels, including individual, social/cultural, built environment, and policy [11]. Such models have provided the conceptual basis for studying environmental correlates of physical activity and other health-related behaviors because these environmental factors can affect large populations over long periods of time [12], and can provide an empirical basis for interventions. In this context, research linking the built environment with physical activity has increased rapidly in recent years [10,13-15], and is now an international priority [16,17].

In the developed countries of North America, Australia and Europe, consistent findings have emerged that people who live in neighborhoods with supportive built environment features like high residential density, street connectivity, proximal access to destinations, good aesthetics and availability of infrastructures and facilities are more physically active than those living in neighborhoods with unsupportive environmental features [14-16,18,19]. Africa is the only continent for which evidence on the association between the built environment and physical activity is lacking [16,20,21], and questions remain about the applicability of surveys constructed in developed countries to the local contexts in Africa [22]. To address this problem, there have been recent calls for investigators in Africa to adapt built environment measures to African contexts [17,21,22]. If evidence- based environmental interventions are to be developed in the African region, there is the need to first tailor environmental measures to African countries and then use the adapted measures to identify promising environmental correlates of physical activity in these countries. Understanding environmental correlates of physical activity and other health behaviors in Africa has implications to inform international evidence-based and country-specific physical activity policies and interventions to help prevent obesity and other chronic diseases [21]. Such interventions would not only help reduce the effect of growing epidemics of inactivity in developing countries [23], but also contribute to effective global prevention of non-communicable diseases that are high in developed countries and growing rapidly in low and middle income countries [1-3,5]. However, valid measures of theoretically relevant environmental variables applicable to Africa are needed for research to progress and for the development of an applicable evidence base in African countries.

The Neighborhood Environment Walkability Scale (NEWS) is a frequently used questionnaire for assessing perceived attributes of the neighborhood environment for physical activity [24-28]. The NEWS is being used by the International Physical Activity and Environment Network (IPEN) for cross-country analyses of built environment and physical activity relationships [21,29]. It is a reliable and valid instrument [24,26,30-32], that has been tested internationally and translated into many languages [18,25,26,32-34]. However, the NEWS was developed in the USA, and its applicability to other environments may be limited due to differences in culture and environmental features [32,35]. African urban environments are different from those in the developed countries. Most African urban neighborhoods have diverse terrains, land use, infrastructures, transportation and road designs that may not be adequately captured by measures constructed in the developed countries. While prevalence of attributes characterizing a Western lifestyle like high car ownership, dog walking and having pets appear to be lower in African countries like Nigeria, high crime rates including assault, rape, robbery, burglary and motor vehicle theft are more prominent compared to western developed countries [36]. Though adaptation of surveys is essential there is value in retaining items and constructs that will allow for comparisons of findings across countries [21]. Therefore, adaptation and evaluation of environmental measures constructed elsewhere is required as a first step before such measures can be used to adequately evaluate built environments in Africa.

Since a major goal of IPEN is to represent the worldwide variation in built environment, psychometric evaluation of NEWS in sub-Saharan African countries would allow for international and cross-national comparison of findings on environmental correlates of physical activity research. It is particularly important for research in this field of study to establish measures of both internationally comparable environmental variables and those of particular relevance to the local environment in Africa. Because Nigeria is the most populous country in Africa with culture and languages similar to most sub-Saharan African countries, it is a good choice to evaluate the feasibility of using the NEWS for measuring built environment in this country. NEWS-Nigeria has potential for advancing physical activity research on environmental correlates of physical activity in other African countries. The aim of the present study was to systematically adapt the NEWS to Nigeria and evaluate aspects of reliability and validity of the adapted NEWS among adults in Nigeria.

## Methods

### Procedures

Participants were recruited from two cities (Maiduguri and Ilorin) in Nigeria. Maiduguri is the largest and capital city of the state of Borno, located in North-Eastern Nigeria and about 920 km North of Abuja, the capital city of Nigeria. The city of Maiduguri has about 749,000 inhabitants and Borno state covers an area of 72,609 km^2^ with a population density of about 57 inhabitants per km^2^[37]. The state of Borno shares boundary with Cameroon, Chad and Niger Republic. Ilorin is the largest and capital city of the State of Kwara, located in the Central part of Nigeria and about 485 km South of Abuja. The city of Ilorin has about 782,000 inhabitants and the state of Kwara covers an area of 35,705 km^2^ with a population density of about 66 inhabitants per km^2^[37]. Both Maiduguri and Ilorin have neighborhoods located in the inner city and Government Reserved Areas (GRA) or new layout areas that have a diversity of housing patterns, land use mix and access and street characteristics. Consistent with high walkability neighborhood characteristics [33,38,39], neighborhoods in the inner city in Maiduguri and Ilorin have a high concentration of multiple family residences, non- residential land uses (small retail stores, shops, local markets and places of worship) and streets with short block length with many alternative routes to destinations. Neighborhoods in the GRA/new layout areas are characterized by predominantly single family homes, few non- residential land uses and streets with longer block length with fewer alternative routes to destinations, consistent with low walkability neighborhood features [33,38,39]. However, the inner city areas in both cities are mostly low socioeconomic status (SES) areas, while the GRA/new layout areas are mostly high SES [40].

A purposive sample of adults was drawn from both cities to represent the diversity of neighborhood environments. In Maiduguri, participants were recruited from eight previously identified neighborhoods (four each in the inner city and GRA/new layout) that have been stratified into high-walkable/low SES and low-walkable/high SES for research purposes [41]. In Ilorin, participants were recruited from 6 non-adjacent neighborhoods that are different in walkability/SES categories (three each in the inner city and GRA/new layout) as identified by the research team (ALO, AYO) and a local urban planning expert. Specifically, the walkability components, defined as residential density, mixed land use and connectivity from previous studies [33,38,39], were used to identify the high- and low-walkable neighborhoods used for participants’ recruitment in the present study. The research team and the expert made summary judgments based on direct observation, local knowledge and professional background in assigning neighborhoods into the two walkability categories.

Sample size was determined using a more conservative effect size (effect size statistic [d=] 0.45) than that found in a previous study [24]. We determined that 77 participants from each neighborhood stratum (high- walkable/low SES [inner city] and low- walkable/high SES [GRA/new layout]) in each city was needed to detect a moderate to large effect size with more than 80% power [42]. Recruitment continued until approximately 80 individuals from each neighborhood type (low and high walkability) in each city had completed the survey and had provided physical activity data (Table 1).

**Table 1:** Descriptive characteristics of the sample (N = 386)

	**Maiduguri sample (n = 280)**	**Ilorin sample (n = 206)**	**Statistics**	**p-value**
Gender (n,%)			1.09†	0.308
Female	90 (50.0)	92 (44.7)		
Male	90 (50.0)	114 (55.3)		
Age (years) Mean (± SD)	35.6 ± 10.3	36.7 ± 3.9	1.16‡	0.248
				
Marital status (n,%)			0.49†	0.537
Married	106 (58.9)	114 (55.3)		
Not married	74 (41.1)	92 (44.7)		
Tribe/ethnicity (n,%)			307.15†	<0.001*
Hausa/Fulani	21 (11.7)	5 (2.4)		
Igbo	8 (4.4)	5 (2.4)		
Yoruba	10 (5.6)	193 (93.7)		
Kanuri/Shuwa	44 (24.4)	0 (0.0)		
Other	97 (53.9)	3 (1.5)		
				
Educational level (n,%)			31.92†	0.001*
>Secondary School	111 (62.7)	149 (72.7)		
Secondary School	38 (21.5)	55 (26.8)		
<Secondary School	28 (15.8)	1 (0.5)		
Occupation (n,%)			3.99†	0.136
White collar	72 (40.0)	70 (34.3)		
Blue collar	45 (25.0)	70 (34.3)		
Unemployed	63 (35.0)	64 (31.4)		
BMI Prevalence (n,%)			6.18†	0.103
Underweight	14 (7.8)	8 (3.9)		
Normal weight	107 (59.4)	142 (68.9)		
Overweight	51 (28.3)	52 (25.2)		
Obese	8 (4.4)	4 (1.9)		
BMI (Kg/m^2^) Mean (± SD)	23.8 ± 3.9	23.8 ± 2.7	0.01‡	0.995
Income (Naira) (n,%)			0.32†	0.957
<15,000	49 (33.1)	59 (32.2)		
16,000-45,000	50 (33.8)	62 (33.9)		
46,000-90,000	30 (20.3)	41 (22.4)		
>90,000	19 (12.8)	21 (11.5)		
Neighborhood type (n,%)			0.04†	0.849
Low SES/high walkable	90 (50.0)	105 (51.0)		
High SES/low walkable	90 (50.0)	101 (49.0)		

†_ Values based on Chi-Square Statistics.

‡_ Values based on independent t-tests.

*- Significant difference between samples (*p* < 0.05).

BMI- Body Mass Index.

Contact was attempted with 225 individuals in Maiduguri (108 in the inner city and 117 in the government reserved areas/new layout). Among the individuals contacted, 80 in both the inner city and government reserved areas/new layout neighborhoods (respectively 74.1% and 68.4%), completed and provided a usable test-retest survey. In Ilorin, 259 individuals (125 in the inner city and 134 in the GRA/new layout) were contacted for the study, and 105 (84.0%) in the inner city and 101 (75.4%) in the GRA/new layout areas provided completed and usable surveys. All participants in the two cities were recruited directly from their homes in the identified neighborhoods. Eligibility criteria included: (1) living within the identified neighborhood categories in the last 12 months, (2) being 20–65 years old, (3) not having any disability that prevented independent walking and (4) being able and willing to complete a written survey in English language, which is the official language in Nigeria.

To assess test-retest reliability, only participants in Maiduguri city were invited to complete the adapted NEWS questionnaire twice, with an interval of two weeks. Test-retest reliability could not be conducted in the city of Ilorin due to logistical difficulty of recontacting participants. Construct validity was assessed by comparing scores on the NEWS among residents living in neighborhoods that differed in walkability in both Maiduguri and Ilorin, similar to approaches in prior studies [24,33]. Concurrent validity was studied by investigating the relationships between NEWS measures and outcomes from the International Physical Activity Questionnaire (IPAQ)-long form. Although transport-related and leisure-time self-reported walking are theoretically hypothesized to be associated with variables assessed in NEWS and are often used as the validity criterion for NEWS measures [18,30], leisure time moderate-to-vigorous physical activity (MVPA), total MVPA and total physical activity also were included as criterion variables for concurrent validity in the present study because guidelines for sufficient health-enhancing physical activity are often based on leisure time MVPA and total physical activity [43-45]. It is therefore important to understand how the NEWS measures relate to various physical activity outcomes of adults in an understudied region like Africa.

Demographic characteristics of participants were obtained on the first day of contact. All participants provided informed consent, and the study was approved by the ethics committee of the University of Maiduguri Teaching Hospital, (ADM/TH/EC/75).

### Measures

#### Neighborhood environment walkability scale

The neighborhood environment walkability scale (NEWS) was adapted to assess neighborhood environment characteristics relevant to sub-Saharan Africa. The research team (ALO, JFS, AYO) and a group of public health scientists from Africa and the IPEN center in the United States coordinated the adaptation of the NEWS for use in Nigeria. The adaptation process was similar to that utilized for the Nigerian physical activity neighborhood environment scale [46] and the Assessing Levels of Physical Activity (ALPHA) questionnaire in Europe [35], and was based on empirical literature from the transportation planning and urban planning fields [38]. Since the goal of the adaptation was to retain as many original concepts and items as possible but to express them in ways that are appropriate for the local culture and environment in sub-Sahara Africa, the final adaptation of the NEWS included all items of the original instrument [24] in their original or slightly modified form, with 18 additional items describing features of the environment relevant to sub-Sahara Africa (see Table 2).

**Table 2:** Nigerian-NEWS: answer frequencies, mean scores, and test-retest reliability scores (N = 386)

	**Answer frequencies and mean score of each item on first assessment (%)**						**Test-retest reliability scores**	
	Types of residences							
Item/scale	None	A few	Some	Most	All	Mean (SD)	Agreement%	ICC
1. Residential density								0.66
a. detached houses	17.6	25.1	13.7	28.0	15.5	2.9 (1.3)	87.3	0.93
b. 1–3 floor town houses with private kitchen	44.3	40.2	10.9	4.7	0.0	1.8 (0.8)	92.1	0.95
c. 1–3 floor apartments with shared kitchen	29.6	14.3	14.0	37.1	4.9	2.7 (1.3)	84.9	0.90
d. 4–6 floor apartments	46.2	27.5	9.1	15.8	1.3	2.0 (1.1)	74.9	0.56
e. 7–12 floor apartments	61.8	10.6	21.0	6.5	0.0	1.7 (1.0)	77.6	0.52
f. >13 floor compounds	40.0	49.1	10.4	0.5	0.0	1.7 (0.6)	82.7	0.75
	1-5 min	6-10 min	11-20 min	21-30 min	>30 min			
2. Distance to facilities (Land Use Mix Diversity)								0.87
a. grocery shop	38.3	29.0	15.0	10.9	6.7	2.2 (1.2)	69.0	0.86
b. supermarket	2.8	4.1	11.4	26.9	54.7	4.3 (1.0)	66.2	0.46
c. electronic shop	4.9	11.9	22.6	29.9	30.6	3.7 (1.2)	60.0	0.74
d. building materials shop*	3.1	8.9	25.7	28.3	34.0	3.8 (1.1)	66.2	0.66
e. food market	13.5	44.0	19.2	11.9	11.4	2.6(1.2)	73.3	0.74
f. laundry/dry cleaners	14.5	31.6	22.8	13.7	17.4	2.9(1.3)	62.8	0.73
g. clothing store	19.9	33.2	21.5	8.8	16.6	2.7 (1.3)	65.5	0.67
h. post office	0.5	1.6	1.8	11.4	84.7	4.8 (0.6)	85.6	0.40
i. library	0.3	0.0	2.1	10.6	87.0	4.8 (0.4)	84.4	0.48
j. elementary school	20.3	41.3	21.0	12.7	4.7	2.4 (1.1)	55.1	0.67
k. other schools	10.1	47.9	21.5	14.5	6.0	2.6 (1.0)	60.0	0.68
l. book store	0.8	2.3	7.0	22.8	67.1	4.5 (0.8)	75.1	0.29
m. fast food restaurant	1.3	2.8	3.4	18.1	74.4	4.6 (0.8)	81.2	0.47
n. ATM/bank	2.1	7.5	10.1	21.6	58.7	4.3 (1.1)	61.1	0.66
o. non fast food restaurant	6.8	13.3	36.8	26.1	17.0	3.3 (1.1)	55.1	0.63
p. food canteen*	19.5	25.5	19.5	16.4	19.0	2.9 (1.4)	70.0	0.77
q. video store	18.9	24.9	30.6	14.8	19.9	2.7 (1.2)	56.1	0.54
r. pharmacy	26.8	42.3	22.1	5.5	3.4	2.2 (0.9)	71.6	0.78
s. saloon/barber shop	21.0	42.1	16.6	9.9	10.4	2.5 (1.2)	71.1	0.78
t. work place	7.1	6.1	10.3	15.9	60.6	4.2 (1.3)	73.4	0.60
u. bus stop/train stop	22.8	30.6	21.0	18.7	7.0	2.6 (1.2)	72.3	0.67
v. taxi/motor bike*	29.3	37.6	20.7	10.6	1.8	2.2 (1.0)	71.8	0.65
w. park	2.1	5.2	13.5	24.4	54.9	4.3 (1.0)	82.8	0.37
x. open field/school field*	13.0	50.4	15.1	13.2	8.3	2.5 (1.1)	58.4	0.65
y. recreation center/sport center	1.3	1.8	4.1	7.0	85.8	4.7 (0.7)	82.8	0.15
z. gym/fitness	1.6	2.6	2.8	7.5	85.5	4.7 (0.8)	86.7	0.49
aa. health care clinic*	7.0	24.6	36.5	18.1	13.7	3.1 (1.1)	61.6	0.65
ab. tap/well water*	67.9	17.4	6.7	5.7	2.3	1.6 (1.0)	77.8	0.68
	Strongly disagree	Somewhat disagree	Somewhat agree	Strongly agree				
3. Land use mix access								0.76
a. possible to do shopping at local stores	11.2	14.8	34.9	39.1		3.0 (0.9)	66.1	0.55
b. shops within easy walking distance	14.2	10.9	40.7	34.2		2.9 (1.0)	74.4	0.67
c. many places in walking distance	17.1	23.1	36.3	23.6		2.7 (1.0)	65.6	0.53
d. easy to walk to transit stop	5.7	18.7	37.6	38.1		3.1 (0.9)	71.2	0.74
e. bad roads/poor drainages*	26.2	24.2	18.7	30.9		2.5 (1.2)	66.7	0.80
4. Street connectivity								0.72
							
a. distance between intersections is short	21.0	13.8	35.8	29.4		2.7 (1.1)	73.4	0.72
b. many four-way intersections	26.4	23.1	29.8	20.7		2.5 (1.1)	72.2	0.77
c. many alternative routes	9.6	17.1	29.0	44.3		3.1 (0.9)	67.2	0.55
5. Infrastructure and safety for walking/cycling								0.59
a. sidewalks on most streets	46.9	25.9	20.2	7.0		1.9 (0.9)	84.5	0.80
b. sidewalks well maintained	63.5	28.8	4.4	3.3		1.5 (0.7)	85.0	0.74
c. bicycle/pedestrian trails nearby	61.8	26.0	7.3	4.9		1.6 (0.8)	86.6	0.37
d. sidewalks separated by parked cars	39.8	28.1	21.1	10.9		2.0 (1.0)	80.5	0.53
e. sidewalks separated by grass/dirt strip	32.4	24.6	29.3	13.7		2.2 (1.1)	81.1	0.68
f. safe to ride a bicycle	17.4	18.4	47.3	16.9		2.6 (0.9)	62.8	0.47
g. streets well lit at night	42.6	19.0	25.7	12.7		2.1 (1.1)	72.7	0.72
h. walkers and cyclists easily seen by people in homes	53.9	20.3	13.3	12.5		1.8 (1.1)	73.5	0.87
i. crosswalks and pedestrian signals available	82.1	12.7	3.4	1.8		1.3 (0.6)	91.7	0.05
j. crosswalks are safe	80.8	14.8	3.9	0.5		1.2 (0.5)	89.4	0.01
k. facilities for bicycle use (lanes etc.) available*	62.0	25.5	6.5	6.0		1.6 (0.9)	89.4	0.52
l. walk path on most streets*	6.5	21.0	42.5	30.1		2.9 (0.9)	76.1	0.76
m. walk path not obstructed*	7.5	38.4	30.9	23.1		2.7 (0.9)	68.3	0.53
n. walk path separated from road*	20.0	24.2	35.8	20.0		2.6 (1.0)	73.3	0.70
o. curb cut ramps from sidewalk to road*	46.4	13.7	29.0	10.8		2.0 (1.1)	88.8	0.20
p. have to cross crowded streets to get to places*	53.8	36.4	8.3	1.6		1.6 (0.7)	74.9	0.68
q. islands for pedestrians in middle of major roads *	65.1	18.0	12.2	4.7		1.6 (0.9)	84.4	0.85
r. pedestrian signals give enough time to cross street*	83.4	11.2	3.1	2.3		1.2 (0.6)	96.1	0.02
6. Aesthetics								0.91
							
a. presence of trees	28.0	20.2	23.8	28.0		2.5 (1.2)	75.0	0.85
b. trees give shade	35.0	19.2	27.7	18.1		2.3 (1.1)	74.5	0.83
c. many interesting things to look at	35.0	17.4	25.4	22.3		2.4 (1.2)	71.8	0.81
d. neighborhood is free from litter	29.1	21.0	21.0	28.8		2.5 (1.2)	74.6	0.85
e. attractive natural sights	51.7	26.2	12.5	9.6		1.8 (0.9)	71.1	0.31
f. attractive buildings	22.0	23.3	23.1	31.6		2.6(1.1)	62.8	0.50
7. Traffic safety								0.65
a. much traffic on street I live	52.0	31.9	11.2	5.0		1.7 (0.9)	82.0	0.74
b. much traffic on nearby streets	39.8	34.1	19.3	6.3		1.9 (0.9)	73.0	0.75
c. speed of traffic on street I live is slow	13.3	21.6	29.7	35.4		2.9 (1.0)	78.0	0.80
d. speed of traffic on nearby streets is slow	26.0	33.6	22.1	18.2		2.3 (1.1)	70.8	0.76
e. drivers exceed speed limits	28.4	28.4	28.1	15.1		2.3 (1.1)	66.8	0.60
f. a lot of exhaust fumes	40.9	27.1	18.0	14.1		2.1 (1.1)	71.5	0.41
g. cars going across sidewalks make it difficult to walk*	36.5	34.9	19.3	9.4		2.0 (0.9)	74.8	0.68
8. Safety from crime								0.66
a. high crime rate	46.5	23.6	18.2	11.7		2.0 (1.1)	82.1	0.82
b. unsafe to walk during day	55.6	38.2	4.7	1.6		1.5 (0.7)	85.0	0.61
c. unsafe to walk at night	33.0	24.9	20.0	22.1		2.3 (1.1)	69.9	0.68
d. presence of gangs induces unsafe feeling*	43.6	24.7	16.4	15.3		2.0 (1.1)	76.6	0.70
9. Single items								
a. parking near local shops is difficult	25.2	27.5	35.3	11.9		2.3 (0.9)	58.9	0.53
b. streets are hilly	56.0	26.2	10.1	7.8		1.7 (0.9)	91.2	0.48
c. streets do not have many cul-de-sacs	20.7	27.5	21.8	30.1		2.6 (1.1)	70.6	0.66
d. many canyons/hillsides in neighborhood	56.0	22.3	12.4	9.3		1.8 (0.9)	91.6	0.53
e. see and speak to other people while walking in neighborhood	19.7	16.1	34.3	29.9		2.7 (1.1)	64.3	0.81
f. many unattended domestic animals*	30.6	15.3	16.4	37.7		2.6 (1.3)	78.0	0.86

ICC = intra class coefficient.

* Specific African items added to NEWS.

The adapted NEWS survey consists of 83 items that assessed the following perceived environmental characteristics: a) residential density (6 items); b) proximity to non-residential land uses (land use mix - diversity) (28 items); ease of access to nonresidential uses (land use mix - access) (5 items); street connectivity (3 items); infrastructure and safety for walking and cycling (18 items); aesthetics (6-items); traffic safety (7 items) and safety from crime (4 items). Six other items were analyzed as single items: parking near local shops is difficult; streets in the neighborhood are hilly; streets in the neighborhood do not have many cul-de-sacs; there are many canyons/hillsides in the neighborhood that limit number of routes; I can see and speak to other people while walking in the neighborhood; there are many unattended domestic animals in the neighborhood. Calculation of the subscales of the adapted NEWS (hereafter called NEWS-Nigeria) and selection of the single items were based on methods proposed by Cerin and colleagues after a cross-validation of the confirmatory factor analysis structure of NEWS [31]. The 18 new items were added to specific scales, based on the content of the items: 6 items were added to the land use mix diversity scale, 1 item to ‘land use mix access’, 8 items to ‘infrastructure and safety for walking and cycling’, 1 item to ‘traffic hazards’, and 1 item to ‘safety from crime’. The presence of unattended domestic animals in the neighborhood was scored as a single item. After adding these items, Cronbach alphas (α) were calculated for the adjusted scales. The α values ranged from 0.64 (traffic hazards) to 0.86 (safety from crime), showing good internal consistency of the scales. Scoring details and a digital version of the original NEWS can be found on http://sallis.ucsd.edu/measures.

All subscales and single items, with the exception of residential density and land use mix-diversity, were rated on a 4-point Likert scale. Residential density items asked about the presence of various types of neighborhood residences, from single-family detached homes to 13-story or higher apartments (more than 13 apartment/rooms for multiple families), with a response range of 1 (none) to 5 (all). Residential density items were weighted relative to the average density of single-family detached residences (e.g., 7- to- 12 story apartments were considered to be 50 times more person-dense than single family residences), and weighted values were summed to create a residential density subscale score [24,30]. Land use mix-diversity was assessed by the walking proximity from homes to various types of destinations, with response ranging from 1- to 5-min walking distance (coded as 5) to >30-min walking distance (coded as 1). Higher scores on land use mix-diversity indicated closer average proximity (higher walkability).

#### Self- reported walking and physical activity

The long version of the International Physical Activity Questionnaire (IPAQ-long) was used to assess participants’ self-reported physical activity and walking for different purposes. The questionnaire assessed frequency (number of days in the last 7 days) and duration (hours and minutes per day) of physical activity in four domains (work, transportation, recreation and household) and of motorized transport. The IPAQ was used to compute weekly minutes of walking for recreation, cycling for transport, walking for transport and leisure time moderate-to-vigorous physical activity (MVPA). Leisure time MVPA was obtained by summing minutes per week of moderate physical activity and vigorous physical activity during leisure-time. Total MVPA was computed by summing the total min/week of reported physical activity of moderate and vigorous- intensities across all four domains. For total physical activity, the total min/week of activities in each domain were summed (total work + total transport + total domestic and garden + total leisure-time min/week scores) to gain an overall estimate of physical activity in a week (http://www.ipaq.ki.se). The IPAQ-long has good reliability (intra-class range from 0.46 to 0.96) and fair-to-moderate criterion validity in a 12-country study (ρ = 0.30), comparable to other self-report measures [47]. Acceptable intraclass correlation coefficients ranging from 0.60 to 0.82 have been reported for the transportation and leisure time physical activity of IPAQ [48], including the walking items used in the present study.

#### Sociodemographic characteristics

Information on age, gender, marital status, religion, income, educational level and employment status were elicited from the participants. Marital status was classified as married or not married. Educational level was classified as more than secondary school education, secondary school education, and less than secondary school education. Employment status was classified into white collar (government, private employed), blue collar (self- employed, trader, artisan etc.) and unemployed (homemaker, student, retired, or unable to find job). Income was categorized into 4 groupings based on NAIRA/month (15 000 NAIRA ≈ 100 US Dollars). Participants’ ethnicity/tribe was classified as Hausa/Fulani, Yoruba, Ibo, Kanuri/Shuwa and other. Height and weight was measured using a digital scale and stadiometer. Body mass index (BMI) was calculated as body weight divided by the square of height (kg/m^2^). The World Health Organization principal cutoff points for BMI were used to create the categories: underweight (< 18.5 kg/m^2^), normal weight (18.5– < 25 kg/m^2^), overweight (25– <30 kg/m^2^) and obese (≥30 kg/m^2^) [49].

### Statistical analysis

Descriptive statistics are presented for the sociodemographic characteristics of all participants. Differences in sociodemographic variables between cities and neighborhood walkability/SES were examined with independent *t*-test procedures for continuous variables and with Chi-square tests for dichotomous variables.

To evaluate the test-retest reliability of the adapted NEWS-Nigeria, one-way model single-measure intraclass correlation coefficients (ICC) were calculated to test individual items. To test NEWS scale scores computed from multiple items, the single-measure ICC was also computed. ICC represents the proportion of total variance in a set of values that is attributable to between-subjects variability, with the remaining proportion attributable to error. ICC estimates >0.75 were considered as good reliability scores, between 0.50 and 0.75 as moderate reliability and <0.50 as poor reliability [50]. Proportion of agreement was also calculated to measure the proportion of occasions that individuals gave the same score. Proportion of agreement above 0.70 was considered high [51].

To assess the construct validity of NEWS-Nigeria, general linear model procedures were used to conduct multivariate ANCOVA tests (adjusting for residents’ age, gender, income, and educational level) to analyze neighborhood differences (high SES/low walkability vs low SES/high walkability) in environmental scales. To assess the concurrent validity of NEWS-Nigeria, Pearson correlations were calculated between environmental variables (scales and single items) and IPAQ measurements. All analyses were performed using IBM SPSS Statistics 19.

## Results

The descriptive characteristics of the sample (N = 386) are reported in Table 1. No differences were found between the samples from both cities for gender, age, marital status, occupation, body mass index, income and neighborhood types. Significant differences were found for education, with more participants with higher education from Ilorin than Maiduguri (*p* < 0.001). The prevalence of overweight or obesity in both cities was comparable with the Nigerian population (23.9%– 38.6% overweight or obese, on population level) [52]. However, participants from both cities were somewhat higher educated compared with the Nigerian population (34.3% with higher education, on population level) [52]. The unemployed were over-represented in both cities’ sub-samples, compared with the unemployment distribution of the general population in Nigeria (19.7%, on a population level) [37].

### Test-retest reliability

Table 2 shows the response frequency and mean score of each item on the first assessment of the adapted NEWS and its test-retest reliability scores. The ICCs of the sum scores of each of the eight subscales ranged from 0.59 to 0.91. Three ICCs (land use mix diversity, land use mix access and aesthetics) were higher than 0.75, indicating good reliability. The five other ICCs (residential density, street connectivity, infrastructure and safety for cycling, traffic hazards, and safety from crime) were between 0.59 and 0.72, indicating moderate reliability. ICCs of the individual items ranged from 0.01 to 0.95 with the lowest scores for particular items of the scale ‘infrastructure and safety for walking and cycling’. In total, reliability of 23 items was good, reliability of 43 items was moderate and reliability of 17 items was poor. However, the poor reliability scores were probably due to a lack of variance in the answers, as the proportion of agreement for the 17 items with low reliability was generally high (>0.70 for 15 out of 17 items with low ICC reliability). Overall, proportion of agreement for all individual items ranged from 55.1% to 96.1%.

### Construct validity

Results of the MANCOVA tests (Table 3) showed that adults living in high-walkable neighborhoods perceived higher residential density, more land use mix diversity, higher street connectivity, more traffic safety and more safety from crime. They also perceived higher activity-friendliness for the single items “parking near local shops is difficult”, “I see and speak to other people while walking in the neighborhood”, “streets are hilly” and “there are many canyons/hillsides in the neighborhood” than adults living in low-walkable neighborhoods. However, individuals living in low-walkable neighborhoods perceived their environment to have better infrastructure and safety for walking/cycling and to be more aesthetically pleasing than individuals living in high-walkable neighborhoods, which was expected because low-walkable areas are also higher in SES. Low-walkable neighborhood inhabitants had more favorable scores (i.e. perceptions of an activity-friendly environment) on the following single items: presence of cul-de-sacs and presence of unattended domestic animals. For land use mix access, no differences were found between high-walkable and low-walkable neighborhood residents.

**Table 3:** Construct validity of Nigerian-NEWS differences in environmental perceptions between objective high-walkable and low-walkable neighborhoods (N = 386)

**Environmental perceptions**	**High-walkable/low SES neighborhoods (n = 195) mean (SD)**	**Low-walkable/high SES neighborhoods (n = 191) mean (SD)**	**F**
**Multivariate model**			67.7***
*Environmental scales*			
Residential density	363.0 (110.8)	256.8 (67.8)	72.2***
Land use mix diversity	2.9 (0.4)	2.4 (0.5)	113.9***
Land use mix access	2.8 (0.5)	2.9 (0.6)	1.4
Street connectivity	3.0 (0.9)	2.6 (0.7)	12.5**
Infrastructure and safety for walking/cycling	1.9 (0.3)	2.1 (0.4)	28.5***
Aesthetics	1.7 (0.6)	3.0 (0.6)	294.7***
Traffic safety	2.6 (0.4)	2.3 (0.3)	40.0***
Safety from crime	2.6 (0.5)	2.3 (0.3)	30.3***
*Single items*			
Parking near local shops is difficult	2.7 (0.8)	2.1 (1.0)	29.1***
Streets are hilly	3.9 (1.1)	4.7 (0.6)	58.3***
Streets do not have many cul-de-sacs	2.3 (1.0)	2.9 (1.1)	13.8**
Many canyons/hillsides in neighborhood	3.9 (1.1)	4.5 (0.8)	25.7***
See and speak to other people while walking in neighborhood	3.4 (0.7)	2.1 (1.1)	148.2***
Many unattended domestic animals	3.5 (0.8)	1.7 (1.0)	286.6***

All analyses were adjusted for age, gender, income and educational level of the participants.

All environmental scales were positively scored, i.e. higher score = more activity-friendly.

** p < 0.01, *** p < 0.001.

### Concurrent validity

Table 4 shows the correlations between the different environmental perceptions (scales and single items) and physical activity (IPAQ). All significant correlations were in the expected direction (i.e. perceived activity-friendliness of the neighborhood was associated with more physical activity), except for the association found between the ‘aesthetics’ subscale and walking for transportation. The single items were not strongly associated with the physical activity outcomes. The only significant items were, ‘I see and speak to other people while walking in the neighborhood’ and ‘there are many unattended domestic animals in my neighborhood’. For the single item ‘presence of domestic animals’, the correlations were not in the expected direction (more unattended animals was associated with more physical activity). The size of all significant correlations ranged between 0.10 and 0.31, indicating low-to-moderate validity. Residential density, land use mix diversity, and traffic safety had significant correlations with most of the physical activity outcomes. As expected, land use mix diversity was more strongly related to transport activity than leisure time activity, and aesthetics was more strongly related to leisure time activity than transport activity. Significant correlations with environmental perceptions were found most often for MVPA and total physical activity.

**Table 4:** Predictive validity of Nigerian-NEWS: Pearson correlations between environmental perceptions and the long last seven day IPAQ (N = 386)

**Environmental scale**	**Walking transport**	**Cycling transport**	**Total transport**	**Leisure time walking**	**Leisure time MVPA**	**Total leisure time PA**	**Total MVPA**	**Total PA**
Residential density	0.11*	0.12*	0.15**	0.02	0.01	0.01	0.21***	0.19***
Land use mix diversity	0.25***	0.12*	0.28***	0.18**	0.08	0.12*	0.28***	0.31***
Land use mix access	−0.01	0.01	−0.01	0.07	0.17**	0.17**	0.06	0.05
Street connectivity	−0.03	0.04	−0.01	0.05	0.16**	0.16**	0.19***	0.18***
Infrastructure and safety for walking/cycling	−0.05	0.06	−0.02	0.11*	0.22***	0.22***	0.13*	0.10
Aesthetics	−0.11*	0.01	−0.10	0.06	0.11*	0.11*	−0.01	−0.03
Traffic Safety	0.11*	−0.01	0.09	0.11*	0.14**	0.16**	0.21***	0.23***
Safety from crime	−0.03	0.12*	0.03	0.09	0.05	0.06	0.15**	0.15**
								
**Single items**								
Parking near local shops is difficult	0.08	−0.06	0.05	−0.08	0.01	−0.02	0.05	0.08
Streets are hilly	0.01	−0.01	0.01	−0.01	−0.04	−0.04	−0.07	−0.08
Streets do not have many cul-de-sacs	−0.01	0.02	−0.02	−0.03	−0.06	−0.06	0.01	0.02
Many canyons/hillsides in neighborhood	0.01	−0.03	0.01	−0.02	−0.08	−0.07	−0.07	−0.07
See and speak to other people while walking in neighborhood	0.14**	0.01	0.13*	0.07	−0.03	0.01	0.15**	0.18**
Many unattended domestic animals	0.26***	0.03	0.24***	0.06	−0.09	−0.05	0.15**	0.21***

PA = physical activity, MVPA = moderate-to-vigorous physical activity.

All environmental scales were positively scored, i.e. higher score = more activity-friendly.

* p < 0.05, ** p < 0.01, *** p < 0.001.

## Discussion

This study evaluated aspects of reliability and validity of the adapted NEWS measure in Nigeria. The findings generally indicated acceptable test-retest reliability and construct validity, and low-to-moderate concurrent validity for subscales of NEWS-Nigeria. The test- retest reliability values (ICC = 0.59 to 0.91) reported for the NEWS subscales in the present African study are comparable to those reported for the original version in the USA (ICC = 0.58 to 0.80) [24], the Australian version (ICC = 0.62 to 0.88) [33] and the Chinese translation of the abbreviated version (ICC = 0.57 to 0.99) [25]. Comparable to the pattern of findings in the USA and Australian studies, the subscales on aesthetics, and walking and cycling infrastructures, demonstrated respectively the highest and lowest reliability coefficients in the Nigerian version of NEWS. It is possible that NEWS items that pertain to aesthetics and pleasantness of the environments are ubiquitously more stable than those that focus on neighborhood infrastructures for walking and cycling, regardless of developmental pattern and differences across countries. Perhaps there are ambiguities in what constitutes a sidewalk and bicycle facility, as well as their qualities, that contribute to lower reliability of the infrastructure scale.

Very low reliability coefficients were found for some of the items of the Nigerian version of NEWS: “curb cut ramps from sidewalks to road”, “crosswalk and pedestrian signals are available”, “pedestrian signals gives enough time to cross street” and “crosswalks are safe”. These items assessed features of infrastructures and safety for walking and cycling that are not common in most Nigerian neighborhoods. Although the uncommon items may be omitted when using the adapted NEWS in Nigeria, it is recommended to retain these items as part of the Nigerian version because items like pedestrian signals can become indicators of progress that would be difficult to measure in future if these items were deleted. Retaining these items is also essential for comparing these attributes across countries. The little variability observed in the responses to these items from our sample likely accounted for the low reliability for the items. Nevertheless, the majority of the items on the Nigerian version of NEWS, including most of the newly-developed items, demonstrated moderate to high reliability coefficients, comparable to those found in other studies [18,24,25,33].

Statistically significant differences were found in the ratings of environmental features between residents living in high- and low-walkable neighborhoods for all the subscales (p < 0.001) of NEWS-Nigeria except for land-use mix access, indicating that residents from neighborhoods with different characteristics in Nigeria do perceive their environments differently. Because the neighborhoods were selected to be environmentally different based on the criteria of observed residential density, land use mix and street connectivity, finding that residents of the high-walkable neighborhoods perceived environmental attributes that focus on residential density, land use mix diversity and street connectivity better than residents of low-walkable neighborhood provides evidence of construct validity for NEWS-Nigeria. This result is consistent with findings from the developed countries where these traditional components of walkability are usually rated higher by residents of high-walkable neighborhoods than those of low-walkable neighborhoods [24,33].

Unlike in studies from western developed countries where traffic safety and safety from crime were often not rated differently between neighborhoods [24,33], residents of high-walkable neighborhoods in this African study significantly perceived their neighborhood to have better traffic safety and to be more safe from crime than residents of low-walkable neigborhoods. This finding seems important because the high-walkable neighborhoods in the study were low SES, and still people perceived more traffic and crime safety in these neighborhoods than the low-walkable/high SES neighborhoods. This pattern could suggest differences in expectations about traffic safety and safety from crime between residents of low and high walkable neighborhoods in Nigeria. It is possible that people from high SES neighborhoods could be more ‘demanding’ about what is safe for them. However, this finding could just be a reflection of the general and pervasive insecurity in Nigeria. Perhaps, insecurity from traffic and crime was more pronounced to those living in the low-walkable/high SES neighborhoods than those in high- walkable/low SES neighborhoods at the time of the study.

It is not surprising that low-walkable neighborhoods had higher scores on the subscales on aesthetics and infrastructures for walking and cycling. Low walkable neighborhoods in Nigeria are high in SES and are often found in GRA/new layout areas with more facilities (paved and maintained sidewalks and roads, streets well lit at night) for walking and cycling, and generally have better aesthetic qualities (scenic view, attractive buildings, free from litter) compared to high walkable neighborhoods that are often found in inner cities (low SES areas) with limited infrastructures for walking and cycling, and poor aesthetic qualities [46]. Overall, the significant differences found for the subscales and all single items of the adapted NEWS, suggested the measure is sensitive to discriminate between different neighborhood environment attributes in Nigeria, and provide strong support for its construct validity [24,33].

The mean values for land-use mix access, street connectivity and traffic safety in both the high- and low- walkable neighborhoods in the present Nigerian study were comparable to those in the studies conducted in the USA [24] and Australia [33]. This finding perhaps, reinforces the suggestion that some consistency exists in the use of the NEWS to measure walkability attributes between neighborhoods across metropolitan areas in different countries [33]. It is generally expected that housing patterns, transportation infrastructures and stage of urban development in Africa will be different from those in the western high income countries. Therefore, finding that the mean values for residential density in both neighborhood types were higher in Nigeria, and the mean values for land use mix diversity, infrastructure for walking and cycling, and aesthetic qualities (high-walkable neighborhood only) were lower in the present study compared to the USA [24] and Australian [33] studies is not surprising. African urban environments are generally denser in terms of population, have congested streets with a lot of air pollution, have limited infrastructures for active transportation and may be less aesthetically pleasant compared to those of the developed high income countries.

Most scales on NEWS-Nigeria had correlations in the expected direction with multiple physical activity outcomes. Residential density, land use mix diversity, and traffic safety were correlated with the most physical activity measures. Consistent with domain-specific models of physical activity [12,53], land use mix diversity was more highly correlated with active transport and aesthetics was more highly correlated with leisure time activity in the present study. Walking and cycling infrastructure had inconsistent correlations across physical activity domains in the literature [10,13-15], but in the present study was correlated with the active leisure domain. Contrary to expectations [24], street connectivity was correlated with leisure activity instead of transport activity. Previous studies from Nigeria have also reported inconsistent associations of street connectivity with physical activity outcomes [22,54]. Perhaps, the concept of street connectivity as espoused in the international literature is not an important component of neighborhood walkability relevant to active transportation in Nigeria.

The crime safety scale has an inconsistent record of association with physical activity in the literature [10,13-15], but it was correlated with total physical activity outcomes in the present study. Three previous studies from Nigeria consistently found perception of neighborhood safety from crime to be positively associated with both objective moderate-to-vigorous physical activity, self-reported walking, and body weight status [22,41,55]. Thus, evidence is accumulating that crime safety perceptions are an important correlate of physical activity among Nigerian adults.

An important finding was that most of the scales and two of the items on NEWS-Nigeria were relatively strongly correlated with leisure time MVPA, total MVPA and total physical activity. Because guidelines for sufficient health-enhancing physical activity are often based on leisure time MVPA and total physical activity [43-45], these findings indicate that perceived activity-friendliness of the neighborhood as assessed by the subscales of NEWS-Nigeria was associated with more health-related physical activity. This is somewhat consistent with recent findings from developing countries that favorable environmental constructs including those assessed by the NEWS are associated with more leisure time MVPA, total MVPA and total physical activity [56-58]. These findings suggest neighborhood environment features are important to health behaviors of adults in developing countries. However, the relatively strong associations of most NEWS subscales and items with total MVPA and total physical activity in the present study could be due to the influence of confounding factors like inclusion of occupational and household activities in estimates of total MVPA and total physical activity. Occupational and household activities are likely higher in the high walkable neighborhoods that are typically of low SES in the current study. Thus, correlations of NEWS-Nigeria scales with total PA and MVPA may be overestimated.

Although the correlations of the adapted NEWS variables with physical activity were often significant, they were low. However, observed correlations were comparable with other studies that assessed the NEWS and other environmental questionnaires [18,30,59]. For example, in a study of US adults, significant correlations of NEWS subscales with walking for transport and walking for leisure varied from 0.08 to 0.29 [27], which was comparable to the range of 0.11 to 0.28 with walking for transport and leisure in the present study. Thus, correlations of neighborhood environment variables and physical activity are surprisingly similar in the US and Nigeria. Replicating this pattern of findings in Nigeria supports a conclusion that some environmental constructs assessed by NEWS are generalizable across continents. Perhaps some NEWS subscales are able to assess environmental variables in relation to physical activity similarly across countries regardless of developmental patterns and cultural differences. Findings such as these have international implications for utility and robustness of NEWS measures as important for identifying promising environmental variables that could be manipulated for improving physical activity and controlling obesity worldwide. Use of adapted versions of the NEWS may be particularly important for research in low-income and developing countries that do not have geographic information systems databases that would allow objective measures of environmental attributes.

The present study has some important limitations. First, conducting the study among residents of two neighborhood types from only two cities in Nigeria may restrict environmental variability. Restricted variability could underestimate the strengths of environment-physical activity associations in environmental studies [16,29]. Second, the modest sample size and the non-probability nature of the sample may reduce generalizability of findings. A third limitation was that neighborhood SES in the two cities was confounded with walkability, making it impossible to conclude that the environmental differences are independent of SES. Although individual SES was adjusted for in some analyses, neighborhood level SES can have an important influence on the differences in environmental perceptions between high- and low-walkable neighborhoods. Thus the findings that most environmental subscales assessed by NEWS were associated with more leisure time MVPA, total MVPA and total physical activity in the present study should be interpreted with caution because it is possible that neighborhood SES rather than neighborhood environmental factors accounted for these relatively strong associations.

A strength of the study was the recruitment of participants from two distinct neighborhood types, enhancing heterogeneity in sociodemographics and built environments [53]. Another strength was the systematic adaptation of the NEWS, retaining most of the original items to allow for international comparison while tailoring the measure to reflect Africa-specific conditions. NEWS-Nigeria was developed through regional collaborations similar to those used for the ALPHA questionnaire that was tailored to Europe [35], and was based on empirical evidence and cross-validation of confirmatory factor analysis with existing NEWS measures. The present study supports the feasibility of using NEWS-Nigeria for assessing environmental correlates of physical activity in the African region and can provide leads and guidance to researchers and practitioners in other African countries when evaluating the built environment for health behaviours. Because a major goal of IPEN is to represent the worldwide variation in built environment, conducting the present study in an understudied African region like Nigeria adds to the international literature on worldwide relevance of built environment for promoting physical activity. The present study was the first to report on the psychometric properties of the NEWS in the African region, and suggests the need to create an Africa-wide version of NEWS that can be used within different populations and settings across Africa.

## Conclusion

In summary, this study showed that NEWS-Nigeria has acceptable evidence of reliability and construct validity, and moderate evidence of concurrent validity. This finding is particularly important for health promotion in sub-Sahara Africa because chronic disease rates are rising in the region [1,2,60]. Understanding environmental correlates of physical activity is a priority that could lead to better strategies to prevent further declines in physical activity and support increased physical activity among those already inactive in sub-Sahara Africa. Thus, the development of culturally applicable NEWS measures for Africa could support evidence based interventions against the epidemics of inactivity-related non-communicable diseases in the African region. For example, present findings suggest that higher density, mixed use planning and transportation policies that favor active transport could play important roles preventing non-communicable diseases in Africa, similar to health-related recommendations for more developed countries [7,10,61]. Further adaptation and evaluation of the NEWS in other African countries could lead to evidence-based recommendations for creating better-designed and safer communities that make people more comfortable being physically active in the African region.

## Competing interests

Authors declare there is no competing financial interest associated with this study.

## Authors’ contributions

ALO contributed to conception and design of the study, drafted the manuscript, was responsible for data acquisition and preparation and was involved with interpretation of data. JFS contributed to conception and design and critically revised the drafted manuscript for important intellectual content. AYO was involved with study design, acquisition of data and critically revised the manuscript for important intellectual contents. BD and IDB participated in the design of the study, interpretation of data and critically revised the drafted manuscript for important intellectual content. DVD contributed to study design, conducted the statistical analysis and interpretation of data and helped to draft the manuscript. All authors read and approved the final manuscript.
